# Nursing technicians’ professional training in patient safety: A mixed-methods study

**DOI:** 10.1590/1518-8345.6214.3819

**Published:** 2023-01-30

**Authors:** Ruth Cardoso Rocha, Fernanda Valéria Silva Dantas Avelino, José Wicto Pereira Borges, Agostinho Antônio Cruz Araújo, Maria Augusta Rocha Bezerra, Benevina Maria Vilar Teixeira Nunes

**Affiliations:** 1 Universidade Federal do Piauí, Departamento de Enfermagem, Floriano, PI, Brazil; 2 Universidade Federal do Piauí, Departamento de Enfermagem, Teresina, PI, Brazil; 3 Universidade de São Paulo, Escola de Enfermagem de Ribeirão Preto, Centro Colaborador da OPAS/OMS para o Desenvolvimento da Pesquisa em Enfermagem, Ribeirão Preto, SP, Brazil; 4 Scholarship holder at the Coordenação de Aperfeiçoamento de Pessoal de Nível Superior (CAPES), Brazil.

**Keywords:** Education, Education, Nursing, Education, Nursing, Associate, Nursing, Patient Safety, Licensed Practical Nurses, Educação, Educação em Enfermagem, Educação Técnica em Enfermagem, Enfermagem, Segurança do Paciente, Técnicos de Enfermagem, Educación, Educación en Enfermería, Educación Técnica en Enfermería, Enfermería, Seguridad del Paciente, Técnicos en Enfermería

## Abstract

**Objective:**

to analyze nursing technicians’ training in patient safety.

**Method:**

a convergent parallel mixed-methods study, where qualitative and quantitative elements were concomitantly implemented and equally prioritized, carried out in three technical colleges linked to a federal public institution in the Brazilian Northeast region. In the qualitative phase, semi-structured interviews were conducted with 24 professors and thematic analysis was applied. In the quantitative study, of the survey type, an instrument was used to screen terms about patient safety with 84 students. The results were combined during the general data interpretation, which was based on the Patient Safety Curricular Guide.

**Results:**

two thematic categories emerged: The professors’ understanding about the theme of patient safety in nursing technicians’ training; and Application and projection of the teaching of patient safety in nursing technicians’ training. In the quantitative study, among the 46 screening terms, their identification in the theoretical-practical context predominated in Courses A and C, totaling 36 (78.3%) and 25 (54.3%), respectively. Theoretical teaching stood out in Course B, with 26 terms (56.5%).

**Conclusion:**

professional training of Nursing Technician students has a technical focus and does not fully contemplate patient safety issues in the teaching-learning process and, thus, constitutes a challenge for training institutions and professors alike.

## Resumo

**Objetivo:**

analisar a formação de técnicos de enfermagem em segurança do paciente.

**Método:**

estudo de método misto paralelo convergente, em que elementos qualitativos e quantitativos foram concomitantemente implementados e igualmente priorizados, realizado em três colégios técnicos vinculados a uma instituição pública federal do Nordeste brasileiro. No qualitativo, realizou-se entrevista semiestruturada com 24 docentes e aplicou-se a análise temática. No estudo quantitativo, *survey*, utilizou-se instrumento para rastrear termos sobre segurança do paciente com 84 discentes. A interpretação geral dos dados realizou a combinação dos resultados e balizou-se no Guia Curricular de Segurança do Paciente.

**Resultados:**

emergiram duas categorias temáticas: Compreensão dos docentes acerca do tema segurança do paciente na formação de técnicos de enfermagem e Aplicação e projeção do ensino da segurança do paciente na formação de técnicos de enfermagem. No estudo quantitativo, dos 46 termos rastreadores, predominou a identificação de termos no contexto teórico-prático, nos Cursos A e C, totalizando 36 (78,3%) e 25 (54,3%), respectivamente. No Curso B, destacou-se ensino teórico, com 26 termos (56,5%).

**Conclusão:**

a formação profissional do discente técnico de enfermagem apresenta foco tecnicista e não contempla, integralmente, no processo ensino-aprendizagem, questões de segurança do paciente e, assim, configura-se desafio para as instituições formadoras e docentes.

## Resumen

**Objetivo:**

analizar la formación de los técnicos en enfermería en seguridad del paciente.

**Método:**

estudio de método mixto paralelo convergente, en el que los elementos cualitativos y cuantitativos fueron implementados simultáneamente y considerados con el mismo nivel de prioridad, realizado en tres escuelas técnicas vinculadas a una institución pública federal en el Nordeste brasileño. En el estudio cualitativo se realizó una entrevista semiestructurada a 24 profesores y se aplicó el análisis temático. En el estudio cuantitativo, *survey*, se utilizó un instrumento para detectar términos sobre seguridad del paciente con 84 estudiantes. Para la interpretación general de los datos se realizó la combinación de los resultados y se tomó como base la Guía Curricular de Seguridad del Paciente.

**Resultados:**

surgieron dos categorías temáticas: Comprensión de los docentes sobre la seguridad del paciente en la formación de técnicos en enfermería e Implementación y proyección de la enseñanza de la seguridad del paciente en la formación de técnicos en enfermería. En el estudio cuantitativo, en los 46 términos indicadores, predominó la identificación de términos en el contexto teórico-práctico, en las carreras A y C, con un total de 36 (78,3%) y 25 (54,3%), respectivamente. En la carrera B se destacó la enseñanza teórica, con 26 términos (56,5%).

**Conclusión:**

la formación profesional de los estudiantes de la carrera de técnico en enfermería tiene un enfoque técnico y no considera de forma integral los temas que tienen que ver con la seguridad del paciente en el proceso de enseñanza-aprendizaje, por lo que es un desafío para las instituciones formadoras y para los docentes.

**Highlights**

(1) The professors recognize the importance of patient safety in technical training.

(2) Teaching patient safety has an indirect, punctual and superficial approach.

(3) The students indicate that the theme of patient safety is not fully addressed.

(4) Nursing technicians’ training fails to fully contemplate patient safety.

(5) Teaching patient safety issues still represents a challenge

## Introduction

In the last decades, the issues associated with patient safety became one of the priority subject matters in the health area at the global level. In this context, the World Health Organization (WHO) suggested the reform and inclusion of content on this topic in the curricula of health courses^1^ ^-^ ^2^, culminating in 2011 with the launch of the Patient Safety Curricular Guide for Multiprofessional Health Education (*Guia Curricular de Segurança do Paciente para o Ensino Multiprofissional de Saúde*, GCSPEMS)^3^. Inclusion of this topic in the curricula involves building a safety culture aimed at reducing errors, equipping future professionals to deal with risks and develop skills to strengthen health systems^4^ ^-^ ^5^.

This multiprofessional edition encompasses Dentistry, Medicine, Obstetrics, Nursing, Pharmacy and other professions included in the health team. Although it was developed to be integrated into undergraduate curricula, it can be applied to different cultures and contexts, such as technical Nursing education, based on specific adaptations to the local requirements and parameters, as well as to the students’ resources and pedagogical needs^3^. 

Technical-level Nursing professionals perform almost all their activities in close contact with the patients. It is for this reason that their training needs to be solid, with the required technical and human skills for an adequate development of the professional practice^6^. Direct and constant contact with the patients turns them more susceptible to the occurrence of events associated with errors in the health care practice^7^. 

In Brazil, the first Nursing technical school emerged in the 1960s. Political, social and economic changes during this period exerted a major influence on Brazilian Nursing mobilizations and questions. While nurses were concerned with consolidating this profession as essential for the health sector, the federal government made efforts to increase industrial production and deal with the financial crisis. Therefore, there was the need for an educational reform, which favored creation of the Nursing Technician course^8^. 

There was perpetuation of a scenario in which nursing technicians’ training was associated with an emphatic appreciation of specific knowledge targeted at sustaining the capitalist production mode^9^. Over time, vocational technical education was the object of new and profound changes, with enactment of the Education Guidelines and Bases Law (*Lei de Diretrizes e Bases da Educação*, LDB) (Law No. 9,394/96) and Federal Decree No. 2,208/97, incorporating the proposal to modernize vocational education in the country^10^. Subsequently, the National Curricular Guidelines for Technical-Level Professional Education were instituted, centered on the commitment to the development of professional competencies^11^.

Despite the growing structuring of technical education in Brazil, the specificity of the organization of Nursing in different professional categories and the expressive participation of nursing technicians in the team indicate that the issue of training these professionals is complex, imposing the choice of central aspects that allow understanding training itself^9^, in search of patient care qualification.

Given this situation, it is verified that Nursing technical training in Brazil has shown a number of weaknesses. Nursing technical courses increasingly display extremely fast and low quality training, contributing to the inclusion on unqualified professionals into the labor market^12^. As a limitation, this teaching has presented as a hegemonic approach aimed at carrying out technical procedures^13^, with requirements of the instrumental training principles, guided by a technical educational project and by lack of reflection on the individual/social reality and on action^14^. Training that goes against the need for professional nursing technicians, in addition to having technical skills, to have a technical-scientific framework for the benefit of good care practices, aiming at reducing adverse events^5^.

This very insufficiency related to Nursing technical training is observed in the international context. Costly adverse patient outcomes, including pressure injury, falls with injuries, and urinary tract infections, are less frequent in hospitals with a higher proportion of undergraduate versus technical-level Nursing professional (in some countries, such as United States of America and Canada, called “nurse practitioners”)^15^. In the Brazilian context, a study that evaluated the Pedagogical Project (PP) of technical Nursing courses revealed gaps in the training of these professionals, with an implicit, punctual and disjointed approach to the contents related to patient safety^16^. 

Therefore, the discussion regarding teaching of this topic in the Nursing technical course is urgent for good quality training, so as to exert a direct impact on the health actions. Thus, the following question was selected to guide the research: How is patient safety addressed in nursing technicians’ training? The objective was to analyze nursing technicians’ training in patient safety. 

## Method

### Study design

A convergent and parallel mixed-methods study characterized by the collection and analysis of qualitative (QUAL) and quantitative (QUAN) data, which were equally prioritized (QUAN + QUAL) and kept independent during the analysis process, with combination of the results during interpretation general. The QUAL data were obtained from interviews with the professors and the QUAN data, from applying the questionnaires to the students. Using procedures that implement qualitative and quantitative components concurrently in different samples aims at understanding multiple perspectives on a single issue^17^. By using different samples, it was sought to understand, among other aspects, the contradictions between qualitative and quantitative findings^18^. 

The report followed the recommendations set forth in the Guideline Consolidated Criteria for Reporting Qualitative Research (COREQ)^19^ for the qualitative stage and in Strengthening the Reporting Observational Studies in Epidemiology (STROBE) for the quantitative stage^13^. 

### Locus 

The study was carried out in three technical Nursing courses (Course A, Course B and Course C), at three schools located in three municipalities linked to a federal public institution in the Brazilian Northeast region. The institutions were chosen due to the historical aspects that enabled adaptation to the various changes guided by the Brazilian educational policies. In addition, it was decided to conduct the study in the aforementioned courses, as they were the only ones with Nursing technical training linked to the institution, in addition to having their own curriculum and enjoying credibility in the region. 

Course A was instituted in 1981 and proposes as a training profile nursing technicians capable of identifying problems in the community and making decisions with the multiprofessional team, seeking to see the client/patient as a whole, meeting their needs through care humanized. Course B, implemented in 2006 in the capital city of the state, trains nursing technicians with due competencies and skills to provide humanized care to healthy or sick people. Course C was opened in 2010 and trains nursing technicians for the development of activities inherent to qualification, acting in a reflective, critical and creative way in order to meet the clients’ basic needs.

All three courses are offered in the subsequent modality and are organized into four modules, lasting two years, distributed over 1,800 hours, with 1,200 hours of theoretical-practical classes and 600 hours of supervised internship. 

### Period

The qualitative and quantitative data collection procedures were performed concomitantly from June to December 2019. 

### Population

The qualitative stage was comprised by professors and the participants in the quantitative stage were students attending all three Nursing Technician courses. It was decided to approach different populations, considering that the professors assume the commitment in nursing technicians’ training and, thus, they know the political-pedagogical approach of the course to which they are linked; and, on the other hand, the students can in fact inform and recognize how patient safety is employed, either in theory or in practice, during the training process.

### Selection criteria

The following inclusion criteria were defined for the qualitative stage: being an effective and temporary professor, active in technical courses; with at least six months performance; and responsible for academic disciplines in which one or more terms related to patient safety were found in the curricula, identified in a previous study^16^. The following exclusion criteria were established: professors who did not return contact after three attempts, on different days and times; and those on maternity or sick leave, although there was no need to apply this latter. 

The following was defined a inclusion criterion for the quantitative stage: being a student regularly enrolled from the third period in one of the three courses, considering that, at this stage, contact with the academic disciplines and internships allows for a better position on the training process. Students who suspended enrollment were excluded, as well as those on maternity or sick leave. 

### Definition of the participants

In the qualitative stage, the sample was defined for convenience, according to the professors’ availability for data collection in the study loci. In total, all three courses have 28 faculty members. To recruit the target population, the researchers held face-to-face meetings with the coordination of the courses and, subsequently, with the professors, presenting objectives, methods, risks and intended benefits. During the recruitment phase, four individuals refused to participate in the research due to time unavailability, which totaled a sample comprised by 24 participants. In order to preserve anonymity and observing the ethical issues, code P (Professor) was employed, followed by an Arabic number corresponding to the order in which the interviews were conducted (P1, P2... P24). 

In the quantitative stage, the sample was of the census type, inviting students able to participate. After previous contact with the course coordinators, the researchers held a meeting with the possible participants to present the study proposal and, at that moment, the date, time and place for data collection were agreed upon with those involved. From the universe of 86 possible participants, 84 students comprised the final sample of this stage.

### Instruments used for data collection

A form on sociodemographic and professional aspects, as well as those related to patient safety, was applied in the qualitative stage. The interview script had the following guiding question: How is patient safety addressed in nursing technicians’ training? A pilot test was previously performed with two Nursing professors from other mid-level institutions: such test indicated that some of the script questions needed to be corrected. These interviews did not comprise the final sample data.

In the quantitative stage, a questionnaire was used to characterize the students’ sociodemographic aspects and knowledge about patient safety. A previously validated instrument was also employed, structured in eight domains and 46 items (screening terms), to assess the training context (theory) and performance in the internships (practice), in the context of patient safety^20^. The domains that comprise the instrument are as follows: what is patient safety?; reasons why applying human factors is important for patient safety; effective team; learning from errors to avoid harms; use of good quality methods to improve the assistance provided; medication safety; interaction with patients and caregivers; and prevention and control of infections. The instrument has an adjectival scale to answer in which context they had contact with a screening term: theoretical or practical; only one of them; or none^20^.

This instrument was prepared to assess the curricula of Nursing undergraduate courses. The decision to apply it in technical course curricula was motivated by the analysis of the items that make up the domains that also apply to this level of education. In addition to that, application in this study expands the use possibilities of the aforementioned instrument.

### Data collection

Data collection was in charge of two previously trained researchers: a PhD student in Nursing and an undergraduate Nursing student (Scientific Initiation). Both of them have been developing studies on patient safety for at least two years and were part of the Research Group in Nursing and Health Education and History. 

In the qualitative stage, an approach was first made in order to justify the interviewer’s interest in the topic and, consequently, the drivers to conduct the research. The interviews were conducted in a private space, in a room offered by the HEI, according to the interviewees’ availability and with the presence of at least of the researchers. After prior authorization, they were audio-recorded in a digital voice recorder. The interviews lasted between 20 and 60 minutes. The information was validated in terms of content by the participants who, at the end of the interview, were asked if they would like to add anything to the reports. The data obtained were stored for full transcription with the aid of the Microsoft Word^®^ program. No interviews were repeated.

In the quantitative stage, data collection was carried out in a private classroom, collectively, with delivery of the instruments in a sealed envelope to each student, who answered individually and with a flexible deadline. 

### Study variables

The study variables established from the quantitative stage consisted of eight domains and the respective 46 screening terms that comprise the instrument for evaluating the training context (theory) and performance in internships (practice), in the scope of patient safety: 1. What is patient safety?; 2. Reasons why applying human factors is important for patient safety; 3. Effective team; 4. Learning from errors to avoid harms; 5. Use of good quality methods to improve the assistance provided; 6. Medication safety; 7. Interaction with patients and caregivers; and 8. Prevention and control of infections^20^.

### Data treatment and analysis

The qualitative data analysis was based on the Thematic Analysis framework^21^, consisting of three stages: pre-analysis, data transcribed by the researcher into text files, to assemble the study *corpus*; floating readings, to understand the text based on the study objectives; and their aggregation into topics, with description and representation of excerpts from the *corpus*. The meanings that emerged enabled interpretation, according to the objectives proposed and leaving room for an in-depth discussion^21^. 

The quantitative data analysis was performed in the Statistical Package for the Social Sciences statistical software program (version 21.0). In order to interpret the data related to the number of triggering terms referenced by the students, according to the context in which knowledge about the patient safety contents was obtained, the parameter of values above or below 50% was used to classify the findings of the instrument to identify cognitive and curricular attributes^20^.

Finally, the researchers used the convergent design to compare the findings from qualitative and quantitative data sources. For this purpose, the diverse information was evaluated using parallel constructions for both types of data, which allowed providing validation for each other and creating a solid basis for conclusions about nursing technicians’ training from the perspective of patient safety^17^. Interpretation of the QUAL+QUAN was performed in the light of authors that dealt with training in Nursing and Patient Safety and according to the GCSPEMS guide^22^.

### Ethical aspects

The research was approved by the Research Ethics Committee of the Federal University of Piauí, according to Certificate of Presentation for Ethical Appraisal No. 85911918.8.0000.5214 and opinion No. 2,563.679, in line with Resolutions No. 466/2012 and No. 510/2016. 

## Results

The participants were 24 professors and 84 students from all three Nursing Technician courses. Regarding the professors, most of them stated having five years of professional practice (54.1%), varying from seven months to 27 years and with a mean of 8.82 and a standard deviation of 8.42. The majority (79.2%) indicated effective employment contracts, with 62.5% of the interviewees stating not having participated in continuous training about patient safety. 

With regard to the students, 35.8% were from Course A, 32.1% from Course B and 32.1% from Course C. There was predominance (88.1%) of the age group between 18 and 25 and of the female gender (72.6%), highlighting that 70.2% reported not having participated in extension or research activities on patient safety. Despite this finding, it was verified that 75% of the participants sought diverse information about this theme in the last years. 

The content of the interviews with the professors was organized into two thematic categories: “The professors’ understanding about the theme of patient safety in nursing technicians’ training”; and “Application and projection of the teaching of patient safety in nursing technicians’ training”. At the same time, when filling out the instrument to assess the context of training and work in internships within the scope of patient safety, the students explained distribution of the screening terms in the eight domains previously described in the method. 

These were indicated according to the acquisition source (theory and/or practice or not obtained). According to the results, of the 46 screening terms about patient safety, in Course A, 78.3% were identified in the theoretical-practical context and 21.7% in theoretical teaching. In Course B, 41.3% were predominant in theoretical-practical teaching, 56.5% in theoretical teaching, and 2.2% were unknown to the students. In Course C, 54.3% were identified in theoretical-practical teaching, 43.5% in theoretical teaching, and 2.2% in no context. None of the terms obtained evidence in the practical context alone, as shown in Table 1. 

In the first category of the QUAL study, the professors showed understanding in relation to the patient safety concept, with approximations to the definition established by the WHO. In some statements, this understanding permeates the concrete definition of the term, including more specific aspects, such as minimizing risks, harms and promoting the patients’ well-being, according to the protocols established by the institutions, in addition to highlighting the importance of the multidisciplinary team to maintain patient safety. These characteristics are found in the following statements: *By patient safety, I understand it as the set of actions, norms and protocols that are carried out by the multiprofessional and nursing team, in order to minimize harms that could be avoidable (...). It’s removing the risk for the patient in any environment, whether hospital, outpatient or basic care* (P6)*. Patient safety is an aid whose objective is to minimize harms to the patient to the extent possible (...). By building patient safe, we avoid harms, avoid harms to the patient’s health and contribute to accelerating the recovery process, treatment and rehabilitation of this patient’s health situation, keep this patient more comfortable, establish the bond* (P14)*.*

In the same direction, the results of the QUAN study show that, in Domain 1 (What is patient safety?), the “Patient safety notions” and “Patient-centered care” items were addressed in 89.0% and 92.7% of the cases, respectively, of the theoretical-practical classes in the courses under study, which indicates the students’ consistent approach to conceptualization of the theme. Regarding Domain 3 (Effective team), the “Interdisciplinary/Health team” item, which was also highlighted in the professors’ statements for safe care, obtained investment of classes in approximately 60.2% in the theoretical-practical context (Table 1).

However, in the same domain, the “Errors involving human, environmental and/or organizational factors” item, which can involve use of institutional protocols, was only addressed, on average, in 49.9% of the theoretical-practical classes. In addition, it is interesting to note that, in Domain 5 (Use of good quality methods to improve the assistance provided), both the “Care quality indicators” and the “Quality improvement” screening items were below 60% in the theoretical-practical and theoretical approaches (Table 1). 

The professors considered that Nursing professionals are the most suitable to avoid health care errors, both due to their representativeness in terms of number and to the procedures performed. Another aspect that was emphasized is the concern about the errors disseminated by the mass media, especially relating them to this professional class: *Basically, everything that Nursing does is related to patient safety (...) I think that the patient safety view helps students and professionals a lot to have a humanized perspective and an integral look at the patient (...)* (P1)*. It’s of fundamental importance in technicians’ training and very relevant in all Nursing categories. Therefore, since the university and technical school, we must bring future professionals closer to this theme (...) we see that every day. The media links errors mainly to the name of Nursing (...) Technicians and nurses are the professionals in the greatest number in the health team and who are most able to reduce the issue of harms to these patients and should be emphasized not only in practice, but also in theory* (P21)*.*

Following the same direction, the students indicated that the “Errors/Types of errors” item from Domain 4 (Learning from errors to avoid harms) was addressed in more than half of the theoretical-practical classes (57.3%), despite significant variability between the courses (from 86.7% to 40.7%), as well as the “How to learn from errors” item, whose mean was 56.2% (varying from 83.3% to 33.3% between the courses). With a lower mean, the “Notification about errors” item was indicated by the students as addressed in nearly 49.2% (varying between 70% and 37%) of the theoretical-practical classes. It is even more worrying that the “Blame culture”, an item from Domain 1 and which deals with the omission and/or concealment of errors made in patient care, was only discussed in 19% of the theoretical-practical classes.

In the second category, the professors reported that the topic is treated in a theoretical and/or practical and unstructured way, with some elements related to the subject matter, being taught in only four academic disciplines (Nursing Fundamentals, Older Adults’ Health, Pharmacology and Perioperative Nursing), and following the six patient safety protocols of the National Patient Safety Program (*Programa Nacional de Segurança do Paciente*, PNSP), according to the following statements: *Nursing Fundamentals is a discipline that works directly with patient safety (...). Some time ago we began to have specific patient safety classes, from hand hygiene to biosafety. We point out those items that must be observed, what can be done to prevent harms, I emphasize the protocols and the use of the 25 Do’s, proper administration of medication (...)* (P2)*. In Older Adults’ Health we talk about falls, then I present the clinical case showing pre-exposure to the risks and ask them to find the risks the patient is exposed to (...). During the internship in the Basic Health Unit (BHU), we offer lectures on the prevention of falls and injuries in older adults, on proper medication use, on proper storage (P10). In the Pharmacology discipline, I focus on the drug administration process that must be followed in order to reduce the risk to which this patient will be exposed, given that drug administration is the activity most performed by nursing technicians and nurses, and it’s also the activity that triggers more harms to the patient’s health due to errors (...)* (P15)*. In the Perioperative Period discipline, we have a subject that is Safe Surgeries, which save lives, one of the axes of patient safety, and it is worked on throughout the discipline. For example, even in sterilization, care with the material, the hygiene principles, it can be a contamination link for the patient and a cause for the occurrence of hospital infections (...)* (P17)*.*

The students’ results showed that, in Domain 2 (Reasons why applying human factors is important for patient safety), the “Safety in the use of equipment” item was evaluated as addressed in theoretical-practical classes up to 100% of the times. In Domain 8 (Prevention and control of infections), “Antisepsis” and the “Antisepsis techniques” were also significantly mentioned, with a mean of 93.8% regarding reference in theoretical-practical classes. These topics are commonly addressed in the academic disciplines and in the contents mentioned by the professors, showing convergence between the data.

The professors also stated that the theme of patients’ rights and duties is a frequently alluded subject matter in Nursing technical teaching, mainly in the “Nursing Fundamentals” discipline. These findings diverge from the quantitative stage data, as a reduced percentage of students (36.3%) stated having theoretical-practical knowledge about health system users’ laws and rights.

Although the Pharmacology discipline was mentioned by the professors as a place of allusion to the aspects related to patient safety, with an indication also by the students in the QUAN stage that the “Medication system and drug prescription, distribution and administration processes” item was included in 85.4% of the theoretical-practical classes, it was verified that “medication errors” were represented in less than half of the mentions (45.2%) of the theoretical-practical classes (Table 1). 

According to the professors, although the topic of patient safety has gained space in recent years, the need for a more targeted approach emerges, in order to motivate and include students in these contents, with participation in events dealing with this theme. They also highlighted indispensability of the content in a visible way in the PP, as the importance of the topic is unquestionable, given the statistical data of errors and harms in health care. 

During the interviews, there was a projection of professors regarding issues related to patient safety, emphasizing what can be improved to leverage teaching on the subject matter in nursing technicians’ training. In their statements, the professors highlighted the need to work on the topic more consistently, especially in the theory, as some participants reported that they felt more comfortable exploring this topic in the practice. The professors also agreed that they did not feel properly prepared to conduct this subject matter in the classroom and, therefore, required continuing education in order to get to know and explore the topic with the students. These perspectives are corroborated in the following statements: *Here, at college, we’re not involved in this issue of patient safety training, for these future nursing technicians, and we end up talking more about the practice (...) I think it’s still super deficient (...) in a certain way, it’s not within the PP, course plan and menu (...) it will depend a lot on the professional who understands the importance of working with the students (.. .) we have to evolve in our pedagogical project in relation to this, bringing it into the classroom so that they can see its importance and apply it in the practice* (P8)*. (...) it’s still a new topic for many people. So, if we encourage students to participate in events that discuss this topic, it may be important to reflect on the harms that can be caused to the patient* (P6)*. I think that more epidemiological aspects of the infections in the health services could be worked on, and such aspects should be treated more rigorously, in more depth (...)* (P20)*.*

Differently, when considering the sum of the terms related to patient safety of all three courses, the quantitative analysis indicated that, in general, 58% stood out in the theoretical-practical context, indicating that most students recognize the approach to the topic in these scopes. Even so, it is to be emphasized that some items relevant for safe care presented an incipient approach in the theoretical-practical context, as indicated by the students, namely: pandemic (13.1%); biofilm (15.3%); outbreak (22.8); epidemic (24.9%); community infection (29.0%); healthcare-associated infections (32.5); microbial resistance (36.4%); health system users’ laws and rights (36.3%); isolation (36.6); and conflict resolution (39.9%). On the other hand, aspects involving procedures to be performed by the future nursing technicians, such as hand hygiene (93.9%), antisepsis (93.8%), antisepsis techniques (93.8%), patient-centered care (92.7%) and safety in the use of equipment (90.1%) were significantly mentioned, above 90% (Table 1). 

In the statements, it was observed that the Healthcare-Associated Infections (HAIs) constitute a theme that would need progress, with regard to the training process, and should be treated with more rigor and depth in the nursing technicians’ training, due to the high infection rates. This finding corroborates data from the quantitative study, in which most students reported having only theoretical knowledge (51.5%) on the subject matter, which can refer to the need for them to be better disseminated in the practical activities (Table 1).

**Table 1:** Distribution of the items related to patient safety mentioned by the students attending the Nursing Technician courses, according to domains and screening terms (theory and/or practice and not obtained). Teresina, PI, Brazil, 2020

	Domain/Screening terms	Theoretical-practical (%)			Mean	Theoretical (%)			Mean	Practical (%)			Mean	Not obtained (%)			Mean
		CA	CB	CC		CA	CB	CB		CA	CB	CC		CA	CB	CC	
1	Patient safety notions	96.7	88.9	81.5	89.0	3.3	11.1	18.5	11.0	0	0	0	0	0	0	0	0
	Patient-centered care	96.7	92.6	88.9	92.7	0	3.7	11.1	4.93	3.3	0	0	1.1	3.7	3.7	0	2.5
	Adverse events	80.0	51.9	48.1	60.0	16.7	48.1	33.3	32.7	0	0	3.7	1.2	3.3	0	3.7	2.3
	Errors involving human, environmental and/or organizational factors	53.3	37.0	59.3	49.8	43.3	55.6	25.9	41.6	0	0	0	0	3.3	7.4	14.8	8.5
	Blame culture	20.0	14.8	22.2	19.0	43.3	33.3	14.8	30.5	0	0	0	0	36.7	51.9	63.0	50.5
2	Use of ergonomics principles in care	73.3	59.3	88.9	73.8	26.7	40.7	11.1	26.2	0	0	0	0	0	0	0	0
	Fatigue and stress in professional performance	70.0	51.9	44.4	55.4	20.0	48.1	55.6	41.2	3.3	0	0	1.1	6.7	0	0	2.2
	Safety in the use of equipment	100.0	74.1	96.3	90.1	0	22.2	3.7	8.6	0	0	0	0	0	3.7	0	1.2
	N95 or PFF2	20.0	11.1	33.3	21.5	53.3	55.6	51.9	53.6	0	0	0	0	26.7	33.3	14.8	25.0
	Regulating norm No. 32	23.3	14.8	44.4	27.5	66.7	59.3	55.6	60.5	0	0	0	0	10.0	25.9	0	12.0
	Standard precautions/Use of PPE	96.7	81.5	88.9	89.0	0	18.5	11.1	9.9	0	0	0	0	3.3	0	0	1.1
	Workers’ immunization	93.3	66.7	70.4	76.8	0	33.3	25.9	19.7	0	0	0	0	6.7	0	3.7	3.5
3	Interdisciplinary/Health team	73.3	44.4	63.0	60.2	23.3	55.6	33.3	37.4	0	0	0	0	3.3	0	3.7	2.3
	Effective leadership	53.3	37.0	44.4	44.9	46.7	63.0	55.6	55.1	0	0	0	0	0	0	0	0
	Conflict resolution	56.7	29.6	33.3	39.9	36.7	70.4	51.9	53.0	0	0	0	0	6.7	0	14.8	7.2
	Supervision	83.3	29.6	59.3	57.4	10.0	59.3	40.7	36.7	3.3	7.4	0	3.6	3.3	3.7	0	2.3
	Communication process in the workplace	93.3	74.1	74.1	80.5	6.7	25.9	25.9	19.5	0	0	0	0	0	0	0	0
4	Errors/Types of error	86.7	44.4	40.7	57.3	10.0	51.9	55.6	39.2	0	0	3.7	1.2	3.3	3.7	0	2.3
	How to learn from errors	83.3	33.3	51.9	56.2	16.7	55.6	48.1	40.1	0	0	0	0	0	11.1	0	3.7
	Notification about errors	70.0	40.7	37.0	49.2	30.0	40.7	55.6	42.1	0	0	0	0	0	18.5	7.4	8.6
5	Care quality indicators	43.3	48.1	48.1	46.5	56.7	48.1	51.9	52.2	0	0	0	0	0	3.7	0	1.2
	Improvement in the assistance provided	50.0	55.6	51.9	52.5	50.0	40.7	48.1	46.3	0	0	0	0	0	0	0	0
6	Side effects	93.3	55.6	63.0	70.6	6.7	40.7	37.0	28.1	0	0	0	0	0	3.7	0	1.2
	Medication system and drug prescription, distribution and administration processes	93.3	81.5	85.4	85.4	6.7	18.5	14.8	13.3	0	0	3.7	1.23	0	0	0	0
	Medication errors	46.7	37.0	51.9	45.2	53.3	55.6	40.7	49.9	0	0	0	0	0	7.4	7.4	4.9
7	Health system users’ laws and rights	53.3	18.5	37.0	36.3	46.7	81.5	63.0	63.7	0	0	0	0	0	0	0	0
	Respect for the patient’s health needs	80.0	40.7	74.1	65.0	20.0	59.3	25.9	35.1	0	0	0	0	0	0	0	0
	Family responsibility and interactions in the care provided to the patient	96.7	55.6	88.9	80.4	3.3	44.4	11.1	19.6	0	0	0	0	0	0	0	0
8	HAIs^*^	56.7	11.1	29.6	32.5	43.3	59.3	51.9	51.5	0	0	0	0	0	29.6	18.5	16.0
	In-hospital infection	73.3	37.0	59.3	56.5	26.7	63.0	40.7	43.5	0	0	0	0	0	0	0	0
	Community infection	50.0	11.1	25.9	29.0	50.0	85.2	63.0	66.1	0	0	0	0	0	3.7	11.1	4.9
	Biofilm	20.0	11.1	14.8	15.3	53.3	48.1	59.3	53.6	0	0	0	0	26.7	40.7	25.9	31.1
	Pandemic	13.3	3.7	22.2	13.1	86.7	96.3	74.1	85.7	0	0	0	0	0	0	3.7	1.2
	Epidemic	26.7	7.4	40.7	24.9	73.3	92.6	59.3	75.1	0	0	0	0	0	0	0	0
	Outbreak	16.7	18.5	33.3	22.8	83.3	81.5	66.7	77.2	0	0	0	0	0	0	0	0
	Risk of infection	83.3	33.3	48.1	54.9	16.7	66.7	48.1	43.8	0	0	0	0	0	0	3.7	1.2
	Cross-infection chain	76.7	44.4	44.4	55.2	23.3	48.1	55.6	42.3	0	0	0	0	0	7.4	0	2.4
	Hand hygiene	96.7	88.9	93.9	93.9	3.3	11.1	3.7	6.0	0	0	0	0	0	0	0	0
	Disinfection	96.7	55.6	96.3	82.9	3.3	40.7	3.7	15.9	0	0	0	0	0	0	0	0
	Antisepsis	100	85.2	96.3	93.8	0	14.8	3.7	6.2	0	0	0	0	0	0	0	0
	Antisepsis techniques	100	85.2	96.3	93.8	0	14.8	3.7	6.2	0	0	0	0	0	0	0	0
	Precaution measures and control of the infections	86.7	63.0	92.6	80.8	13.3	37.0	77.4	42.6	0	0	0	0	0	0	0	0
	Isolation	43.3	33.3	33.3	36.6	53.3	59.3	63.0	58.5	3.3	0	0	1.1	0	7.4	3.7	3.7
	Microbial resistance	50.0	25.9	33.3	36.4	46.7	74.1	63.0	61.3	0	0	0	0	3.3	0	3.7	2.3
	Processing contaminated articles	66.7	37.0	70.4	58.0	30.0	55.6	25.9	37.2	0	0	0	0	3.3	7.4	3.7	4.8

* HAIs = Healthcare Associated Infections

## Discussion

Despite being considered a new science, inclusion of the contents on patient safety is still presented as a recent proposal in educational institutions^23^, recognizing that it is challenging to incorporate any new content into a curriculum, especially when it comes to this theme, which requires addressing countless subject matters that are not traditionally taught to students in the health area, such as human factors, systemic reflection, teamwork and error management^4^ ^,^ ^22^ ^,^ ^24^. This situation is verified in performance of the interpretation of the entire analysis, when it is identified that, although the students recognize the allusion to the theoretical aspects about some elements of the topic, other points, of greater complexity and multidisciplinary approach, need to be deepened. 

It is verified that, as in other contexts of Nursing training, patient safety adopts a predominantly technical approach, which can compromise patient safety, which has been challenged by some factors, including lack of creativity, inadequate professional competence, lack of judgment and decision-making ability, insufficient knowledge and skill in care, and lack of effort to improve professional skills^25^. 

Patient safety is relevant in several teaching areas, including Nursing teaching and practice. As well as other members of the health team, technical Nursing have the opportunity of improving patient safety quality. In this sense, professors play a vital role in improving the knowledge required from the students, as well as their attitude and perception towards patient safety. They can ensure that the students are prepared to provide a safe environment and to care for the patients^26^. It is therefore essential to promote students’ knowledge and skills in non-technical issues, including patient safety, in order to increase their ability to deal with the challenges they encounter in clinical environments^27^.

The “blame culture” was identified in the professors’ reports, when they showed concern about the occurrence of errors from the perspective of professional failure, when reporting the fear of errors being broadcast in the media, instead of seeing them as opportunities to improve the system. This teaching attitude was reflected in the results of the quantitative study, where the “Blame culture” item was presented as the most unknown term by the students, confirming that this issue must be explored effectively in the three courses under study. The fact that the students claim not having had the necessary contact with this concept contributes to the continuity of a system of punitive ideas in the face of failures, not giving them the opportunity to understand the error as an opportunity to learn, as well as to develop skills to avoid them^28^. 

The blame culture should be substituted with a fair culture within the care environments. Some health services even discuss errors in an individualized way, in which penalties are applied to the person responsible for an error, which presupposes a false resolution of the problem, triggering various consequences for the professional involved. Errors should be systematically disclosed, motivating the professionals to see them as part of the system and, thus, seek to remove the factors that contribute to that^29^ ^-^ ^30^. 

Application of the patient safety topic in nursing technicians’ training was presented in a superficial and implicit way. The professors identified the need to link the related contents to the theory, as they noticed that it is more worked on in the practical activities. This need was not identified in the quantitative stage, as most students indicated that, in general, the screening terms about patient safety are referenced both in the theoretical and in the practical context.

While there is growing evidence about the need to emphasize explicit patient safety principles in health professionals’ undergraduate education at the universities, there is no evidence of the degree to which this is addressed in technical Nursing (LPN) courses. The knowledge about the extent to which the patient safety principles are addressed in Nursing practical education is incipient^31^.

In their reports, the professors indicated the importance of teamwork, in a multiprofessional modality, and emphasized the nursing technicians’ role in the health team for their performance in terms of safety. Approximations were identified in the quantitative stage, where it was evidenced that the professors applied these concepts with a theoretical-practical approach in the classes. The multidisciplinary aspect is a positive factor in the courses studied, in which teamwork, based on collective action, represents a reciprocal relationship between the multiple technical interventions and the interaction of agents from different professional areas^32^. For this, it is necessary to establish dialog between the members, open communication about errors, and openness for satisfactory interpersonal relationships^33^.

Medication safety was listed by the professors as one of the activities in which they most referred to items related to patient safety, although linked to specific academic disciplines such as Nursing Fundamentals, Older Adults’ Health, Pharmacology and Perioperative Period, showing non-transversality of the topic. The participants cited the strategies for teaching this theme, highlighting the review of the “Do’s” and other measures as an important factor for patient safety, under responsibility of the students during the internships. This emphasis in medication safety had repercussions in the quantitative study, which indicated the consistent theoretical-practical approach of this theme, confirming the importance of this item for safe care.

The professors’ concern in addressing this issue is due to the fact that the errors resulting from the drug therapy represent a growing and challenging problem, in addition to being one of the most common types of incidents in health facilities, which can occur at any of the stages^7^ ^,^ ^34^ ^-^ ^35^. 

A retrospective and correlational study, conducted in collaboration with a system of community hospitals and with the objective of analyzing the relationship between the Nursing team and the occurrence of medication errors, identified that institutions with a lower number of Nursing technicians (internationally called *Licensed Practical Nurses* - LPNs), compared to the number of nurses, present an overall reduction in errors related to drug administration^36^. Such data corroborates the current literature, which shows that the presence of more nursing technicians (LPNs) in the health institution is associated with a higher frequency of adverse events in the patients^15^ ^,^ ^37^. 

The strategies related to the prevention and control of Healthcare-Associated Infections (HAIs) were mentioned by the professors as an aspect that would need to be advanced in the training process, and which must be dealt with in depth in the nursing technicians’ training due to their high rates. This finding corroborates data from the quantitative study, in which most students reported having only theoretical knowledge (51.5%) on the subject matter, which can refer to the need for them to be better disseminated in the practical activities (Table 1). 

The strategies to prevent and control infections should be expanded to all health care settings, and this first and foremost permeates the professionals’ training^38^ ^-^ ^40^. In this direction, the teaching of patient safety, related to infection control, should integrate the curriculum of Nursing courses, as well as be covered by research and extension activities and explored in greater depth, providing practical activities relevant to the professional practice^40^.

Also in this sense, the professors emphasized hand hygiene, associated with the activities developed in the internships and in the specific academic disciplines. In the quantitative study, this screening term was learned by most of the students in the theoretical-practical context. Hand hygiene has proven efficacy in minimizing the microbial load, preventing and controlling cross-transmission by microorganisms, being considered a strong indicator of care quality in terms of patient safety^41^ ^-^ ^43^. Despite the vast dissemination of diverse information regarding effectiveness of hand hygiene as a means to prevent HAIs, adherence is still insufficient, as 70% of the health professionals do not perform the practice following the proper technique and at appropriate times^2^ ^,^ ^44^.

A study conducted with health professionals working in a Neonatal Intensive Care Unit from state of Bahia, Brazil, verified that the team of nursing technicians consisted of the population that presented the lowest adherence rate to hand hygiene^45^. Consequently, it is recommended to adopt efficient and continuing educations training measures, as well as to improve the offer in terms of supplies, in order to promote adherence among these professionals. Therefore, the students attending the technical course should be duly qualified during their training, so that the put proper hand hygiene into practice when they provide care. 

The topic of processing contaminated articles was mentioned by the professors as one of the ways to provide safe care, citing the sterilization principles as fundamental for preventing patient contamination. This item was found in the quantitative study. Thus, it is understood that, in addition to adding quality to Nursing care, good practices in the processing of instrumental articles for health provide safety for the users and professionals involved^2^ ^,^ ^46^. 

The comparison between the context in which the screening terms were cited (theory and practice, theoretical classes and practical teaching) and the course (A, B and C) showed that Course A differed from Course B in relation to both situations. In this sense, Course A presented more screening terms in the theoretical-practical context; and Course B, in the theoretical approach, indicated that it presents teaching in the predominantly theoretical patient safety context. This can have implications for the training of nursing technicians who need effective inclusion in the practical scope for satisfactory curricular integration and, therefore, quality training^5^ ^,^ ^47^ ^-^ ^48^. 

It is relevant to consider the indispensable connection between theory and practice. This means reconceptualizing the education models and processes previously used to develop and assess Nursing professionals’ competence and aptitude for the practice. It is necessary to overcome the theory-practice gap, created by the traditional organization of the practice of professional training in health and, at the same time, help students to transit through the liminal spaces between the orderly and abstract world of technical course classrooms and the real world of the Clinical Nursing practice^49^.

The strengths and limitations regarding nursing technicians’ training for patient safety identified from the expanded analysis developed by this study can contribute to broadening the discussions on the indispensability of consistently and systematized incorporating the theoretical-practical contents about of this theme in technical Nursing education. This fact can effectively contribute to creating and strengthening the patient safety culture, as well as exert a positive influence on good quality care results.

It is considered that this study was limited by the scarcity of research studies that specifically addressed students’ training in technical Nursing courses for patient safety. 

## Conclusion

When analyzing the intersections of professional training in the teaching perspective and student evaluation, it was verified that technical Nursing training comprises insufficient and disjointed approaches to patient safety. Although most of the professors are familiar with patient safety concepts and understand the relevance of this subject matter in the training process of nursing technicians, there is a need to recognize the weaknesses inherent to this path, by indicating an indirect, punctual and superficial approach to the topic, generating direct consequences in training. 

On the other hand, in a movement of convergence and some divergences with the teaching perspective, the students also recognize that the technical Nursing courses did not cover the topic in its entirety. Although the professors did not criticize the technical and procedural training that focuses on safe care aspects only related to the know-hows, the students indicated a limited approach that fails to address the comprehensive training needs of the nursing technicians’ work in the Brazilian reality, involving, in addition to technical implementation, planning and development of reflective, comprehensive and, consequently, safe care. 

Aspects of a merely theoretical approach or that have not been identified need to be improved, so that they are known and explored by the students, with emphasis on the absence of an approach to the blame culture. Nursing technicians’ training showed not to fully contemplate patient safety issues in the teaching-learning process, representing a challenge for institutions and professors alike. 
